# Home Textile Pattern Emotion Labeling Using Deep Multi-View Feature Learning

**DOI:** 10.3389/fpsyg.2021.666074

**Published:** 2021-04-19

**Authors:** Juan Yang, Yuanpeng Zhang

**Affiliations:** ^1^School of Textile and Clothing, Nantong University, Nantong, China; ^2^Department of Medical Informatics, Nantong University, Nantong, China

**Keywords:** home textile pattern, emotion labeling, deep learning, multi-view learning, feature selection

## Abstract

Different home textile patterns have different emotional expressions. Emotion evaluation of home textile patterns can effectively improve the retrieval performance of home textile patterns based on semantics. It can not only help designers make full use of existing designs and stimulate creative inspiration but also help users select designs and products that are more in line with their needs. In this study, we develop a three-stage framework for home textile pattern emotion labeling based on artificial intelligence. To be specific, first of all, three kinds of aesthetic features, i.e., shape, texture, and salient region, are extracted from the original home textile patterns. Then, a CNN (convolutional neural network)-based deep feature extractor is constructed to extract deep features from the aesthetic features acquired in the previous stage. Finally, a novel multi-view classifier is designed to label home textile patterns that can automatically learn the weight of each view. The three-stage framework is evaluated by our data and the experimental results show its promising performance in home textile patterns labeling.

## Introduction

Emotion is the spiritual essence of home textile design. Fabric pattern is an important component of home textiles, which contains rich emotional information, including aesthetics and values. Therefore, fabric patterns rich in connotation and emotion are more and more respected by designers, which can meet the multiple needs of consumers. However, the pattern materials in home textile design and production are increasing day by day, and there are tens of thousands of patterns in the sample database. It is difficult for designers to make full use of the existing rich fabric patterns for home textile design. Therefore, how to integrate the objective characteristics (color, texture, pattern, etc.) and perceptual experience of fabric patterns into a mathematical model for aesthetic evaluation, emotional classification and retrieval, and emotion labeling of fabric patterns is one of the important topics for computer vision and textile design researchers.

With the continuous development of computer science, AI (artificial intelligence) and CV (computer vision) provide ideas and methods to solve this problem. Gan et al. (2014) made use of deep self-taught learning to obtain hierarchical representations, learn the concept of facial beauty, and produce human-like predictor. Datta and Wang (2010) established the first image aesthetics scoring website ACQUINE in 2010. Although the accuracy of the evaluation results is not high, it has shown that calculable aesthetics is feasible. Li and Chen (2009) adopted the features of color and composition in artistic aesthetic features to realize the classification of high and low aesthetic sense of paintings, and achieved a classification accuracy of more than 70%. Lo et al. (2012) studied image aesthetic classification from the aesthetic perspective of image color, layout, edge, and other features, and the results showed that image aesthetic features could be used for image sentiment analysis. With the development of deep learning, many deep learning based methods are also used for image aesthetic classification. Lu et al. (2015) considered both the local and global perspectives of images, designed the CNN (convolutional neural network) model for feature learning and classifier training, and evaluated the aesthetic quality of 250,000 images from AVA database. Compared with the traditional manual feature methods, this method has a great improvement in classification performance. Dong et al. (2015) used the CNN model trained by ImageNet large physical classification database to extract image features and classify high and low aesthetic values. The effectiveness of this method was proved in two image quality evaluation data sets. The above CNN-based aesthetic classification methods all use the pixel values of sample images in the large database as the input of CNN, without integrating the existing mature manual features. It is often very difficult to obtain a large number of home textile design patterns. Using relatively small and limited image aesthetic data sets for training will easily to lead to problems of overfitting and unstable convergence.

In this study, to achieve home textile emotion labeling, we propose a multi-view learning framework that contains three main components. The first component is used to extract initial multi-view features from the shape, texture, and salient region perspectives. The second component is used to extract deep features from the initial multi-view features by CNN. The last component is used to collaboratively learn from multi-view deep features.

## Data And Methods

### Data

**Figure 1:**
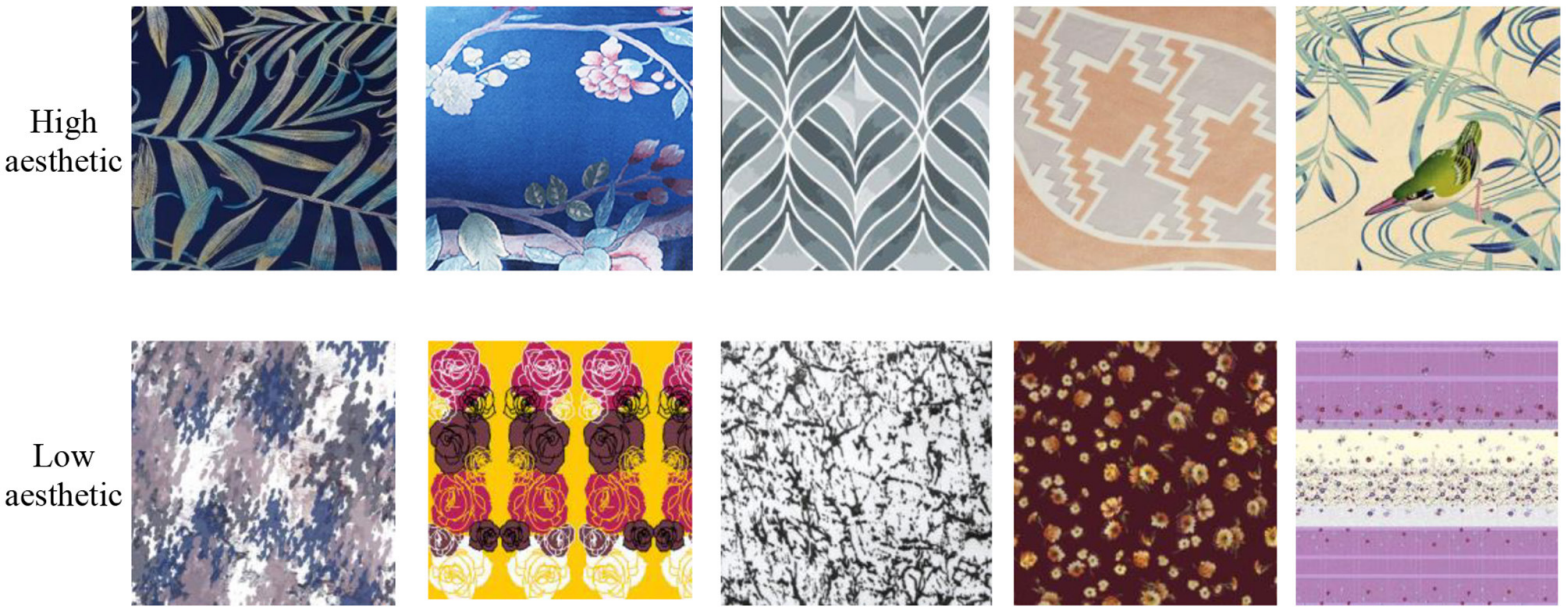
An example of high and low aesthetic home textile patterns.

We employed 20 students from the School of Textile and Clothing of Nantong University to collect 5000 home textile patterns from the Internet. All images were re-sized into 256^*^256 for further use. We also invited another 10 students to conduct subjective aesthetic evaluation on these collected patterns. Students calibrated all the images from three aspects of high aesthetic feeling, low aesthetic feeling, and uncertainty. When the emotion evaluation labels with eight or more participants were the same, this label was determined as the final label of the pattern. After removing the uncertain labels, we finally obtained labeled 4,480 patterns. Sample images and the number of sample images of high and low aesthetic are shown in Figure 1.

We used image clipping and horizontal flipping to enhance the patterns, and intercepted 4 corners and the middle image for mirror processing. After data enhancement, there are 25,000 patterns in each class (high aesthetic patterns and low aesthetic patterns).

### Ethics

The studies involving human participants were reviewed and approved by ethics committee of Nantong University.

Written informed consent to participate in this study was provided by the participants.

### Methods

**Figure 2:**
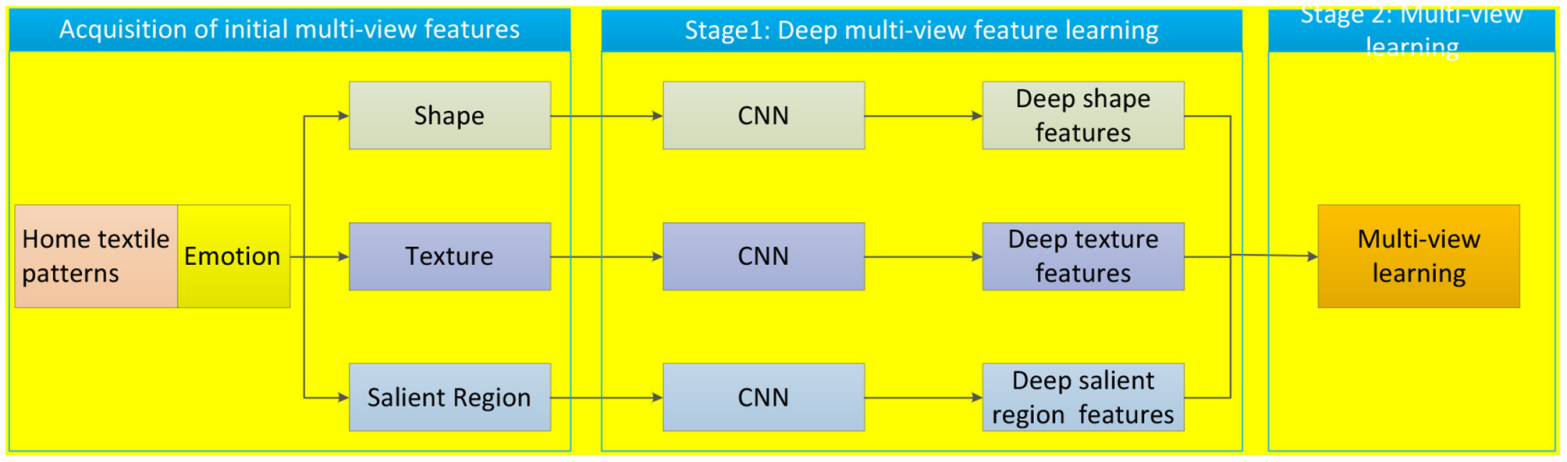
Three-stage framework of emotion labeling.

In this study, we construct a three-stage method for home textile emotion labeling. The first stage is to extract initial shape features, texture features, and salient region features from home textile patterns. The second stage is a CNN-based feature extractor that is used to extract deep features from different aesthetic views from original home textile patterns. With deep features, in the second stage, we design a multi-view classifier to realize emotion labeling. The three-stage framework of emotion labeling is shown in Figure 2.

#### Acquisition of Initial Multi-View Features

**Figure 3:**
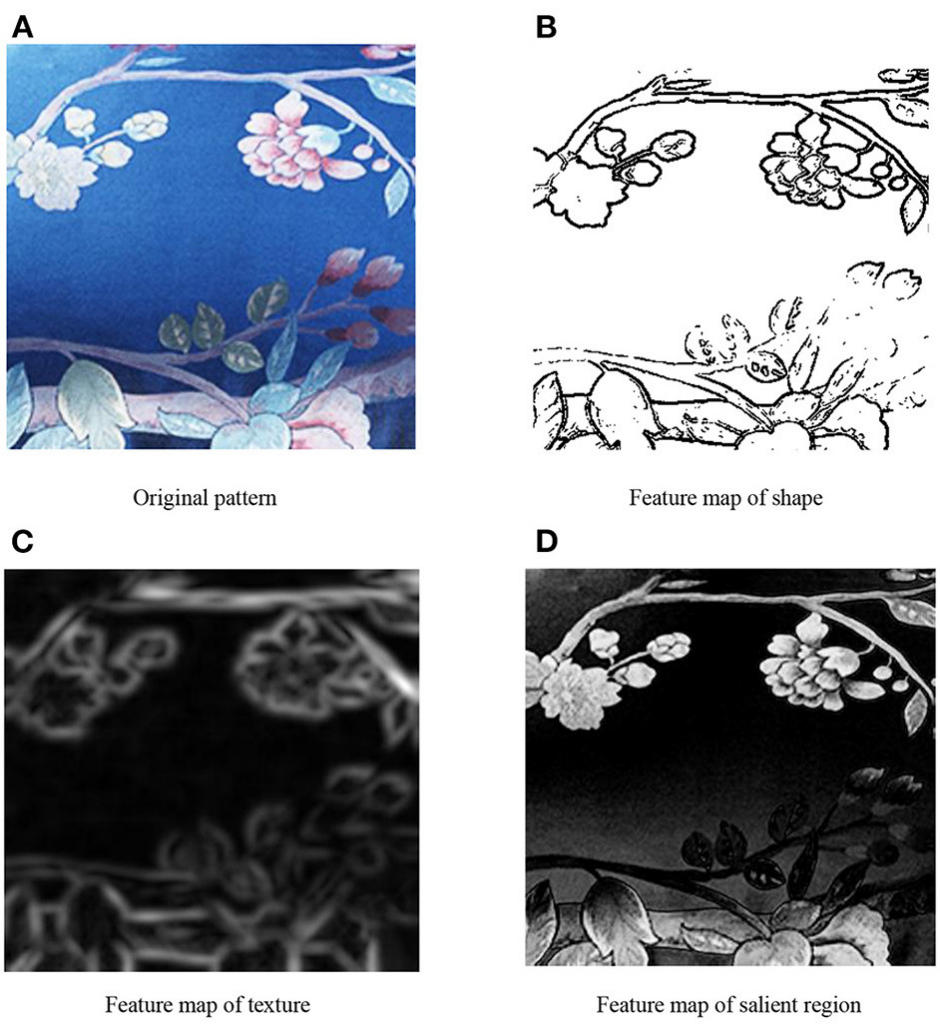
An example of original multi-view features. **(A)** Original pattern, **(B)** Feature map of shape, **(C)** Feature map of texture, and **(D)** Feature map of salient region.

As shown in Figure 2, the initial aesthetic features of home textile contain shape features, texture features, and salient region features. Shape is one of the main characters of design pattern, which can be described by the edge. The edge of an image is a collection of points where the gray value is discontinuous or the gray value changes dramatically. In this study, we use the Sobel operator (Gao et al., 2010) to detect the edge of an image. Texture is a very important content in fabric pattern, which contains many aesthetic features that affect the sense of beauty. The frequency and direction of Gabor filter (Mehrotra et al., 1992) are similar to that of human visual system, which is suitable for image texture feature description. Therefore, in this study, Gabor features are used to represent texture features. The saliency region of an image is the region that attracts the most visual attention and has a more significant influence on the aesthetic feeling of the image. The saliency value of a pixel is defined by the contrast between the pixel and other pixel in the image. Pixels of the same color have the same salience. In this study, we use the LC (Luminance Contrast) (Zhai and Shah, 2006) algorithm to extract salience region features. Figure 3 illustrates an example of initial multi-view features.

#### Deep Multi-View Feature Learning

**Figure 4:**
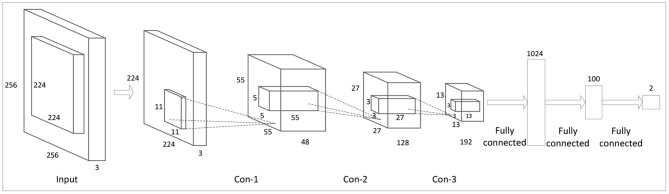
CNN-based deep feature extractor.

In this study, CNN (Sainath et al., 2013; Kalchbrenner et al., 2014; O'Shea and Nash, 2015; Albawi et al., 2017) is employed to extract deep features from home textile patterns from different views. The initial features in shape, texture, and salience region are first constructed following the methods discussed in section ‘Acquisition of Initial Multi-View Features’. The network structure of our deep feature extractor is shown in Figure 4.

1. Input layer: in this layer, we reduce each original three-channel image of 256^*^256^*^3 size to 10 images of 224^*^224^*^3 size by random cropping.
2. The first convolution layer (Con-1): in this layer, the input images are reduced into 55^*^55 feature maps by 48 convolutional kernels (the kernel size is 11^*^11 and the step size is 4). Because the response result of ReLU (Rectified Linear Units) is unbounded (it can be very large), normalization is required. Here, LRN (Local Response Normalization) (Robinson et al., 2007) is used to perform local response normalization.
3. The second convolution layer (Con-2): in this layer, we use 128 convolutional kernels (the kernel size is 5^*^5 and the step size is 2) to further extract features from the 48 feature maps (the kernel size is 27^*^27) generated by the last layer.
4. The third convolution layer (Con-3): in this layer, we use 192 convolutional kernels (the kernel size is 3^*^3 and the step size is 1) to generate 192 feature maps (13^*^13).

Our feature extractor uses the final output to calculate the approximation error and performs back propagation to update the network parameters during training. When the training process is done, the output of the penultimate layer is selected as the deep features learned by our feature extractor. Therefore, the deep features we extracted not only have lower dimensionality than the original features but also possess better discrimination ability to enhance the generalizability of the subsequent classification model.

#### Multi-View Learning

In this section, we will develop a multi-view classifier (Jiang et al., 2016; Qian et al., 2018) for emotion labeling based on the deep multi-view features that are extracted by the CNN-based deep feature extractor shown in Figure 2. The basic idea is that the Shannon Entropy is introduced to the Ridge Regression (SERR) model to automatically identify the weight of each view. With view weights, the deep shape features, deep texture features, and deep salient region features are combined to achieve collaborate learning. Suppose *X*_*k*_ represents the training feature set of the *k*th view, *Y* represents the emotion label of the training set, then the objective function of the new classifier SERR is formulated as follows:

(1) $$\begin{array}{l} J=\overset{K}{\underset{k=1}{\sum }}\omega_{k}\|X_{k}W_{k}-Y{\|}^{2}+\gamma \overset{K}{\underset{k=1}{\sum }}\|W_{k}{\|}^{2}+\delta \overset{K}{\underset{k=1}{\sum }}\omega_{k}\text{ ln }\omega_{k}\\ \begin{array}{l} s.t.\;\overset{K}{\underset{k=1}{\sum }}\omega_{k}=1 \end{array} \end{array}$$

where ω_*k*_ represents the weight of the *k*th view, *W*_*k*_ represents the corresponding transformation matrix, and γ and δ are two parameters to control the penalization terms. The objective function in Equation (1) can be solved by introducing Lagrangian multipliers. To be specific, by introducing the Lagrangian multipliers τ, the corresponding Lagrangian function can be formulated as

(2) $$\begin{array}{l} L=\overset{K}{\underset{k=1}{\sum }}\omega_{k}\|X_{k}W_{k}-Y{\|}^{2}+\gamma \overset{K}{\underset{k=1}{\sum }}\|W_{k}{\|}^{2}+\delta \overset{K}{\underset{k=1}{\sum }}\omega_{k}\text{ ln }\omega_{k}\\ \begin{array}{l} +\tau \left(\overset{K}{\underset{k=1}{\sum }}\omega_{k}-1\right) \end{array} \end{array}$$

By setting $\frac{\partial L}{\partial W_{k}}=0$ and $\frac{\partial L}{\partial \omega_{k}}=0$, we have

(3) $$\begin{array}{l} W_{k}={\left(\omega_{k}{\left(X_{k}\right)}^{T}X_{k}+\gamma I\right)}^{-1}{\left(X_{k}\right)}^{T}Y \end{array}$$

(4) $$\begin{array}{l} \omega_{k}=\frac{\text{exp}\left(-\|X_{k}W_{k}-Y{\|}^{2}/\delta \right)}{\overset{K}{\underset{t=1}{\sum }}\text{exp}\left(-\|X_{t}W_{t}-Y{\|}^{2}/\delta \right)} \end{array}$$

With *W*_*k*_ and ω_*k*_, we can use the alternate iteration to search for the optimal solution. The detailed steps of home textile emotion labeling using deep multi-view feature learning are shown as in Algorithm 1.

**Algorithm 1:** Model training

**Input**: original image set *D*, emotion label *Y*, parameters γ and δ
**Output**: transformation matrix of each view *W*_*k*_, weight of each view ω_*k*_
Procedure:
1. Use Sobel operator, Gabor filter, and LC to extract initial multi-view features.
2. Use deep feature extractor to extract deep multi-view features, *X*_*k*_.
3. Randomly assign ω_*k*_ under $\overset{K}{\underset{k=1}{\sum }}\omega_{k}=1$.
4. Set *t* = 0.
5. Repeat.
6. Use Equation (2) to update *W*_*k*_ based on current ω_*k*_.
7. Use Equation (3) to update ω_*k*_ based on current *W*_*k*_.
8. *t* = *t* + 1.
9. Until (||*W*_*k*_(*t* + 1) − *W*_*k*_(*t*)|| < ε).

After the training procedure is done, for an unseen textile pattern, we use Algorithm 2 to perform emotion labeling.

**Algorithm 2:** Model testing

**Input**: unseen textile patterns, transformation matrix of each view *W*_*k*_, weight of each view ω_*k*_
**Output**: emotion labels of unseen textile patterns
Procedure:
1. Use the feature index obtained from training to select features from the unseen textile patterns.
2. Emotion of unseen textile patterns can be determined by $$\begin{array}{l} Y^{\prime }=sign\left(\overset{K}{\underset{k=1}{\sum }}\omega_{k}X_{k}^{\prime }W_{k}\right) \end{array}$$

## Results

In this section, we will evaluate our new emotion labeling method from two perspectives, i.e., the effectiveness of the deep feature extractor and the effectiveness of the multi-view classifier.

### Settings

To evaluate the effectiveness of the deep feature extractor, we first reshape the original shape features, texture features, and the saliency region features from two-dimensional matrices to one-dimensional vectors and then introduce several traditional feature selection methods for discriminant feature selection. The settings of the introduced feature selection methods are shown in Table 1.

**Table 1:** Settings of feature selection methods.

**Feature selection methods**	**Settings**
MRMR (Togaçar et al., 2020)	Use default setting recommended by Togaçar et al. (2020)
*l*_21_-norm (Nie et al., 2010)	The regularized parameter is searched from [0.001, 10]
PCA (Karamizadeh et al., 2013)	Use default setting recommended by Karamizadeh et al. (2013)
Relief (Urbanowicz et al., 2018)	Use default setting recommended by Urbanowicz et al. (2018)

Additionally, to evaluate the effectiveness of the multi-view classifier SERR we proposed, we directly concatenate all features from different views and use classic classifiers SVM (Support Vector Machine), KNN (K-Nearest Neighbor), NB (Naive Bayes), and DT (Decision Tree) for classification. The settings of the introduced feature selection methods are shown in Table 2.

**Table 2:** Settings of classifiers.

**Classifiers**	**Setting**
SVM (Joachims, 1998)	The Gaussian kernel is adopted. The kernel width is searched from [10^−5^, 10^5^], the center is searched from [10^−5^, 10^5^], and *C* is set to 100
KNN (Zhang et al., 2017)	*K* is set to 5
NB (Rish, 2001)	Use default setting recommended by Rish (2001)
DT (Myles et al., 2004)	Use default setting recommended by Myles et al. (2004)
SERR	γ and δ are searched from [0.001, 10]

All experiments are conducted on a PC with Intel® Core™ i7-9700 @3.00 GHz Dual, 32G memory, and RTX 2080 Ti. The coding platform is Matlab 2012b.

### Experimental Results

In this section, we will report our experimental results from two aspects. First of all, our deep feature extractor is used to extract deep features from each initial view. For comparison studies, four commonly used feature selection methods MRMR, *l*_21_-norm, PCA, and Relief are also introduced to select discriminant features from each initial view. The Ridge Regression model is taken as the classifier for the classification tasks in each view. Tables 3–5 show the classification results in terms of Accuracy, Sensitivity, and Specificity on each view.

**Table 3:** Classification performance in terms of accuracy.

**Feature selection methods**	**Shape features**	**Texture features**	**Saliency region features**
MRMR	0.9458 ± 0.0030	0.7781 ± 0.0028	0.7517 ± 0.0057
*l*_21_-norm	0.9784 ± 0.0028	0.7558 ± 0.0047	0.7510 ± 0.0047
PCA	0.9555 ± 0.0036	0.7697 ± 0.0027	0.7478 ± 0.0092
Relief	0.9420 ± 0.0014	0.7784 ± 0.0015	0.7149 ± 0.0102
Deep feature extractor	0.9816 ± 0.0021	0.7870 ± 0.0099	0.7579 ± 0.0111

**Table 4:** Classification performance in terms of sensitivity.

**Feature selection methods**	**Shape features**	**Texture features**	**Saliency region features**
MRMR	0.9478 ± 0.0023	0.5541 ± 0.0103	0.5147 ± 0.0101
*l*_21_-norm	0.9578 ± 0.0030	0.5458 ± 0.0088	0.5368 ± 0.0117
PCA	0.9412 ± 0.0026	0.5269 ± 0.0201	0.5429 ± 0.0098
Relief	0.9541 ± 0.0026	0.5578 ± 0.0152	0.5025 ± 0.0100
Deep feature extractor	0.9612 ± 0.0037	0.5670 ± 0.0189	0.5435 ± 0.0125

**Table 5:** Classification performance in terms of specificity.

**Feature selection methods**	**Shape features**	**Texture features**	**Saliency region features**
MRMR	0.9236 ± 0.0036	0.8436 ± 0.0056	0.8178 ± 0.0142
*l*_21_-norm	0.9547 ± 0.0021	0.8268 ± 0.0017	0.8362 ± 0.0073
PCA	0.9632 ± 0.0054	0.8817 ± 0.0026	0.8555 ± 0.0089
Relief	0.9785 ± 0.0023	0.8557 ± 0.0053	0.8521 ± 0.0107
Deep feature extractor	0.9818 ± 0.0025	0.8929 ± 0.0081	0.8617 ± 0.0133

Additionally, to highlight our proposed multi-view learning method SERR, with the deep features from different views, we introduce SVM, KNN, DT, and NB as classifiers for comparison studies. Table 6 shows the classification results in terms of Accuracy, Sensitivity, and Specificity on each view.

**Table 6:** Classification performance of SERR, SVM, KNN, NB and DT.

**Classifiers**	**Accuracy**	**Sensitivity**	**Specificity**
SVM	0.9369 ± 0.0024	0.9257 ± 0.0016	0.9478 ± 0.0042
KNN	0.9478 ± 0.0025	0.9327 ± 0.0019	0.9087 ± 0.0015
NB	0.9368 ± 0.0074	0.9264 ± 0.0015	0.9457 ± 0.0014
DT	0.9698 ± 0.0025	0.9524 ± 0.0036	0.9644 ± 0.0025
SERR	0.9865 ± 0.0014	0.9782 ± 0.0045	0.9654 ± 0.0024

## Discussion And Conclusion

With the improvement of automation of home textile production and design and the increasing number of stored home textile pattern images in enterprises, the traditional retrieval methods can no longer meet the needs of home textile manufacturers. It is necessary to conduct aesthetic evaluation and emotional analysis of home textile pattern, so as to provide better services to enterprises and consumers.

Currently, there are two main ways for home textile enterprises to search home textile design patterns. One is to manually classify and number home textile patterns, which is mainly used for enterprise management. However, a large amount of management storage will cause a waste of resources in all aspects. The other is to establish an image retrieval system for the pre-classified home textile patterns, but it needs to manually classify each pattern. However, it requires human classification of each pattern, which is time-consuming and labor-intensive, and not all home textile patterns can be expressed with keywords or symbols, so it is difficult to meet the different retrieval needs of different searchers.

With the development of AI, especially deep learning, in this study, to achieve home textile emotion labeling, we propose a multi-view learning framework that contains three main components. The first component is used to extract initial multi-view features from the shape, texture, and salient region perspectives. The second component is used to extract deep features from the initial multi-view features by CNN. The last component is used to collaboratively learn from multi-view deep features. We demonstrate our method from two perspectives. From the results shown in Tables 3–5, we see that the features extracted by our deep feature extractor drives the best classifier in each kind of features in terms of Accuracy, Sensitivity, and Specificity, respectively. This superiority indicates that deep features are more discriminant than the initial features obtained in the first stage. Additionally, from Table 6, we see that our proposed multi-view classifier SERR performs better than the traditional classifiers, SVM, KNN, DT, and NB, which means that collaborative learning in multiple feature space is more reliable than direct feature concatenation.

The experimental results show that the emotional labeling method proposed in this study realizes the emotional labeling of home textile patterns, provides an auxiliary retrieval method for consumers who want to buy home textile with certain emotional semantics, provides convenience for enterprises in the production and design of home textile patterns, and can meet the multiple needs of consumers. In our future work, we will consider more kinds of deep features and develop more deep feature extractors for emotion labeling.

## Data Availability Statement

The datasets presented in this article are not readily available because we will further enrich them. Requests to access the datasets should be directed to Yuanpeng Zhang, maxbirdzhang@ntu.edu.cn.

## Ethics Statement

The studies involving human participants were reviewed and approved by Ethics Committee of Nantong University. Written informed consent to participate in this study was provided by the participants.

## Author Contributions

JY and YZ designed the whole algorithm and experiments. All authors contributed to the article and approved the submitted version.

## Conflict of Interest

The authors declare that the research was conducted in the absence of any commercial or financial relationships that could be construed as a potential conflict of interest.
